# Sea Cucumber‐Inspired Double‐Network Biopolymer Hydrogels for Programmable Soft Machines

**DOI:** 10.1002/advs.78047

**Published:** 2026-10-11

**Authors:** Luo Yan, He Xiao, Purohit Prashant K., Vining Kyle H.

**Affiliations:** ^1^ Department of Mechanical Engineering and Applied Mechanics School of Engineering and Applied Science University of Pennsylvania Philadelphia Pennsylvania USA; ^2^ Preventive and Restorative Sciences School of Dental Medicine University of Pennsylvania Philadelphia Pennsylvania USA; ^3^ Department of Materials Science and Engineering School of Engineering and Applied Science University of Pennsylvania Philadelphia Pennsylvania USA; ^4^ Center for Innovation and Precision Dentistry University of Pennsylvania Philadelphia Pennsylvania USA

**Keywords:** bio‐inspired materials, biopolymers, double‐network hydrogels, fatigue resistance, marine‐inspired, programmable mechanics, soft robotics

## Abstract

Engineering biopolymer‐based hydrogels with robust load‐bearing capacity, reversible mechanical programmability, and adaptive actuation remains difficult due to weak physical crosslinking and porous structure. Inspired by the mutable dermis of sea cucumbers, a collagen–alginate double‐network elastomeric (CoDE) hydrogel integrates a covalently crosslinked collagen cryogel scaffold with an ionically crosslinked alginate sacrificial network. This hydrogel system is inherently biocompatible and biodegradable. Compression‐induced scaffold densification and ionic locking enable broad tuning of the compressive response, sustaining programmable peak stresses up to 16 MPa. CoDE exhibits reversible lock–unlock states while fully preserving its peak stress, elastic modulus, and energy dissipation after repeated loading to 85% compressive strain. Cyclic compressive loading mechanically “trains” the biopolymer hydrogel by altering its microstructure. Chemically triggering the release of stored elastic energy in the biopolymer network generates actuation forces. Further, CoDE is tested as a bio‐inspired, externally driven swimmer that can be subjected to large‐scale, repetitive deformation with predictable kinematics that match finite‐element simulations at the device level. The CoDE platform has future applications for building soft‐material machines for adaptive soft robotics, dynamic biointerfaces, and mechanically instructive biomaterials.

## Graphical abstract

Inspired by the mutable dermis of sea cucumbers, the collagen‐alginate double‐network elastomeric (CoDE) hydrogel achieves programmable, recoverable, and functional mechanical behavior. The CoDE hydrogel mimics the mutable stiffness of the sea cucumber dermis through a densified collagen cryogel scaffold that is secondarily reinforced by a dissipative, ionically crosslinked alginate network, while maintaining the intact collagen fiber microstructure under repetitive loading. Cyclic compression structurally trains the biopolymer architecture to amplify ionic actuation. CoDE is a versatile system that can be deployed for building biologically inspired soft‐material machines.

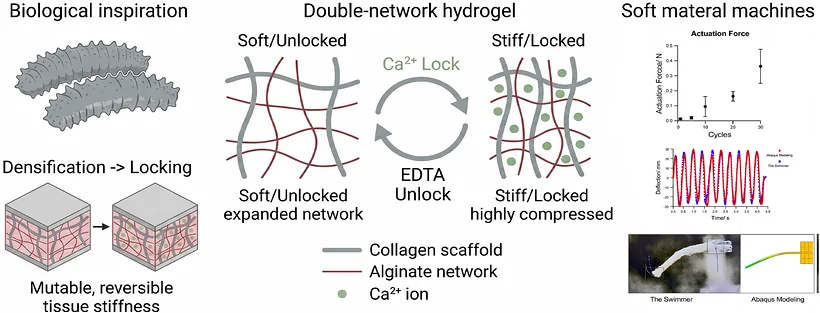

## 1 Introduction

Highly adaptive, reversibly deformable biopolymer systems are widely seen in nature. For example, the dermis of sea cucumbers can rapidly switch between soft and stiff states on demand, enabling them to navigate tight spaces or defend against predators [1]. Fish fins can dynamically change shape to suit different swimming patterns [2, 3]. These examples from nature illustrate biological principles for designing soft, reconfigurable materials that combine toughness with tunability [4] and motivate the development of hydrogel materials for advanced soft machines. Hydrogels feature high water content, biocompatibility, potential for stimulus responsiveness, and the ability to mimic the natural environment of human tissues [5]. There remains a need to develop biopolymer hydrogels that are mechanically robust and reconfigurable, capable of sustaining repetitive, large loads [6].

Dynamic, non‐covalent crosslinks that can break and reform on demand have been used to build reversible polymer systems. For example, a supramolecular hydrogel showed reversible stiffening under acidic, Ca^2+^‐rich conditions [7]. These reversible noncovalent bonds enable self‐healing and reusability. Composite hydrogels mimic the sea cucumber's mutable stiffness by using ionic crosslinking as a reversible switch [8]. However, these previous reversible systems exhibit limited ability to sustain peak stress at the MPa level under large compressive strains. Previous work has demonstrated that double‐network (DN) hydrogels can achieve exceptional toughness by combining an elastic polymer network with a soft sacrificial matrix [9, 10]. We propose extending these concepts to a double‐network hydrogel composed of natural biopolymers that can provide programmability, reversibility, and functional behavior for building soft machines.

Here, we developed the collagen–alginate double‐network elastomeric (CoDE) hydrogel system, consisting of a covalently crosslinked collagen scaffold interpenetrated with an ionically crosslinked alginate network (Figure 1a). CoDE's design is biologically inspired by the dermis of sea cucumbers, which is chemoelastic [11] and can reversibly switch between soft and stiff states to navigate tight spaces or defend against predators [1, 12, 13]. In the CoDE hydrogel, cryogelation is used to generate a densified collagen network that serves as the elastic, load‐bearing backbone [14]. The freeze‐dried collagen structure is rehydrated with alginate biopolymers. Upon compression, the densified collagen scaffold undergoes a phase transition due to progressive buckling in collagen fibers [15] and an increase in the number of contact points between fibers. Here, the phase transition strain also corresponds to the residual strain and is defined as the strain at which the porous scaffold transitions from a rarefied, open‐pore state to a densified, pore‐collapsed state during compression [14]. The collapsed collagen structure is subsequently ionically crosslinked with alginate biopolymers to “lock” the structure and form a sacrificial network that dissipates energy under stress [10]. This hierarchical architecture mimics the sea cucumber's programmable, reversible stiffening mechanism, enabling resilient soft machines with emergent properties (Figure 1b).

**FIGURE 1:**
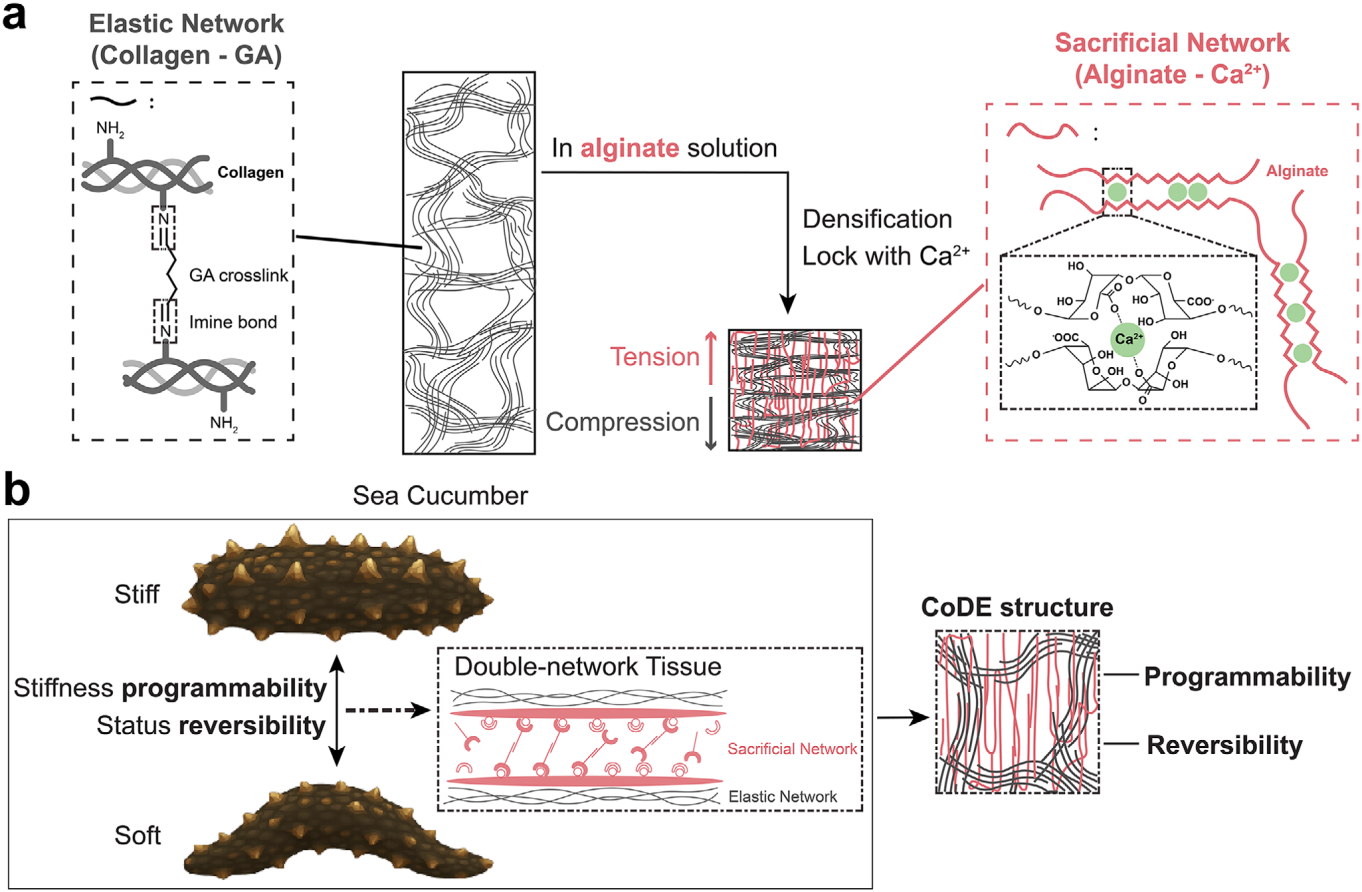
The collagen–alginate double‐network elastomeric (CoDE) hydrogel features a densified collagen scaffold and ionic Ca^2^^+^ crosslinking, enabling programmable, reversible mechanical properties that mimic the mutable stiffness of sea cucumber dermis. (a) CoDE structure is comprised of a glutaraldehyde (GA)‐crosslinked collagen scaffold as the elastic network, which is soaked in alginate solution and densified under compression, followed by Ca^2^^+^ crosslinking to “lock” the compressed collagen network and stretched sacrificial alginate network. The alginate chains are under tension by the restoring force of the compressed collagen scaffold, and therefore, show a more controlled alignment. (b) This system is bioinspired by the collagenous, mutable tissue of the sea cucumber dermis, which provides a model for reversible switching between soft and stiff states. Analogously, CoDE adopts an elastic collagen network coupled with a reversible, sacrificial ionic network to enable stiffness programmability and status reversibility, while maintaining biocompatibility and biodegradability.

## 2 Results

The alginate‐reinforced collagen network of the CoDE hydrogel exhibited programmable mechanical properties that depended on the degree of collagen densification and alginate ionic crosslinking (Figure 2a). CoDE hydrogels were fabricated with tunable collagen densification (0%, 50%, 90%) and Ca^2+^ crosslinking (10 mm and 100 mm). The mechanical performance under repetitive loading was determined by 20 cycles of axial compression in air (Figure 2b,c). The energy dissipated during one hysteresis cycle was defined as the area enclosed by the loading and unloading curves. An example of the CoDE hydrogel loading curve in air is shown in Figure 2d (90% densification with 100 mM Ca^2+^). A 90% compressive strain was used as a large‐deformation condition because the highly porous collagen scaffold (>99.6% porosity [14]) approaches its practical compaction limit after 90% densification. This test further demonstrates CoDE's ability to withstand cyclic large‐scale deformation without fracture. The peak stress stabilized after 5 loops under all conditions (Figure 2e). The CoDE sustained a tunable range of peak stresses from 0.1 to 16 MPa under 90% compression (Figure 2f), spanning a 160‐fold range in compressive strength. Note that the cycles of 90% compression are measured from the new height of the sample after “locking” the densified collagen structure with ionically crosslinked alginate. Energy dissipation per cycle in air increased with densification, particularly under higher Ca^2^^+^ crosslinking (Figure 2g), consistent with the sacrificial, dissipative role of the alginate network. Conversely, the elastic modulus decreased ≈16‐fold (with 100 mm Ca^2+^) with increasing densification level (Figure 2h), likely because the phase transition front easily shifts between the rarefied and densified phases [16]. The peak stress profiles remained comparable across different loading rates (6 mm min^−1^ and 12 mm min^−1^) (Figure S2), indicating a minimal influence of rate‐dependent poroelastic effects.

**FIGURE 2:**
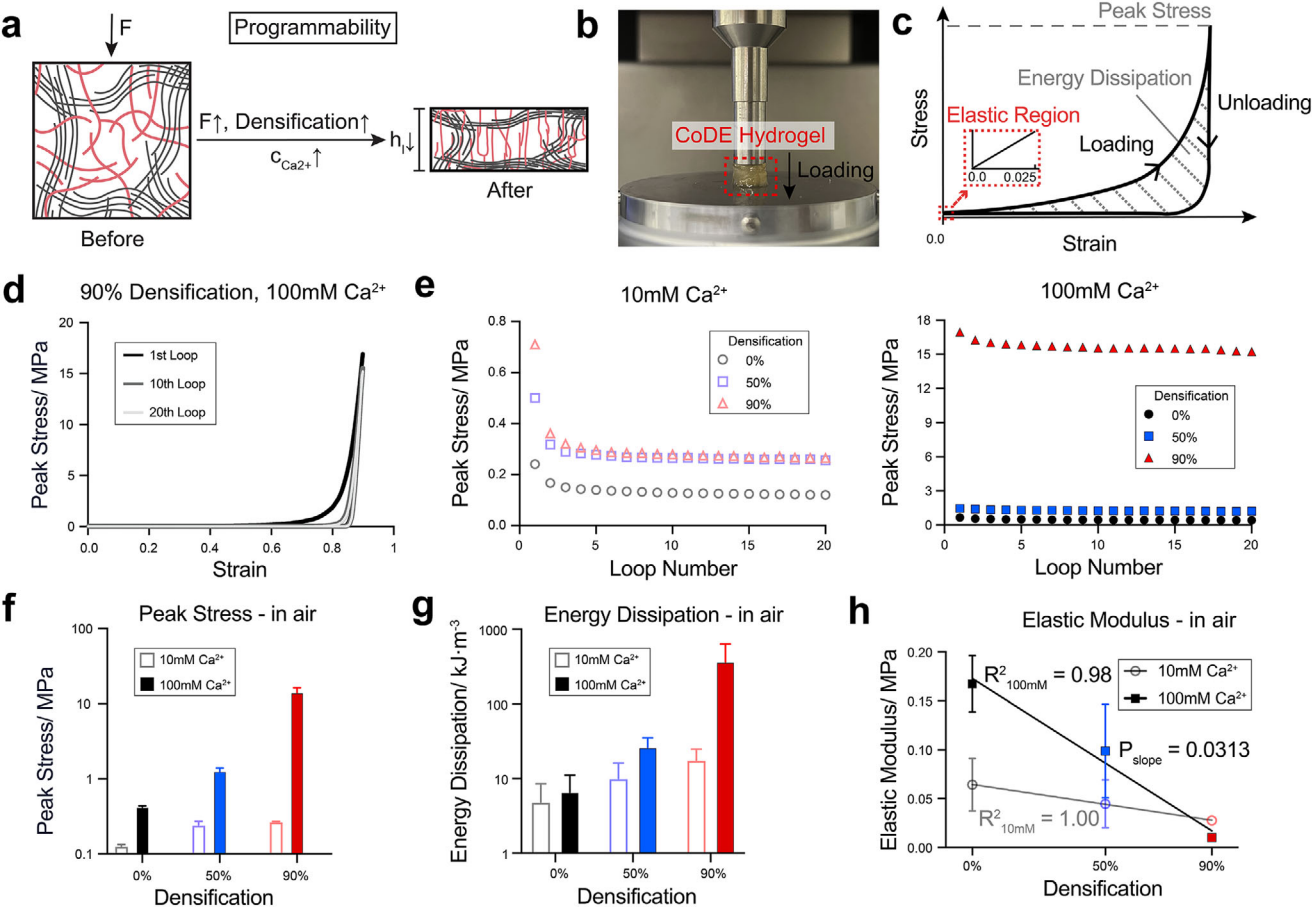
The densified collagen structure of CoDE hydrogels provides programmable hysteresis in loading behavior. (a) CoDE's programmability with densification and locking. (b) Hysteresis test setup for CoDE hydrogel in air. (c) Illustration of the stress–strain curve showing the elastic region (strain: 0–0.025, inset), peak stress, and energy dissipation. (d) 20 hysteresis loops in air for CoDE hydrogel with 90% densification and 100 mm Ca^2+^ crosslinking (among 20 loops, only the first, 10th, and 20th are displayed for simplicity). (e) Peak stress plots of 20 loops of CoDE hydrogels in air (0%, 50%, or 90% densification with 10 mM or 100 mM Ca^2+^ crosslinking): (f) peak stress, (g) energy dissipation, and (h) elastic modulus. Sample size = 3 gels per condition. Error bars show the standard deviation of the mean. Error bars in (h) may appear smaller than the markers.

Next, the reversibility of the CoDE hydrogel was tested by two cycles of locking–unlocking, with 20 hysteresis loops per cycle (Figure 3a). In this work, “locking” refers to compressing the collagen scaffold to a certain densified state and subsequently using Ca^2^^+^ to ionically crosslink the interpenetrated alginate network, thereby fixing the densified collagen structure. “Unlocking” refers to ethylenediaminetetraacetic acid (EDTA)‐mediated chelation of Ca^2^^+^, which dissociates the alginate ionic crosslinks and allows partial recovery driven by the elastic restoring force of the collagen scaffold. No deformation or cyclic loading was applied before the initial locking. An example of a locked and unlocked sample is shown in Figure 3b. The 90% densified collagen structure was locked at a height of 2–3 mm with 100 mm Ca^2+^ crosslinking of the alginate network, loaded, and then subsequently unlocked by chelating the calcium ions with a solution of EDTA. All tests were performed in an aqueous environment (water). Representative stress–strain curves from the 20th loop of cyclic loading to 85% strain show hyperelastic behavior across two locking‐unlocking cycles, with only minor variation in peak stress (Figure 3c). The offset between the end loading point and the start unloading point reflects rapid stress relaxation and hysteretic energy dissipation at the maximum applied strain prior to unloading. The gels partially recovered to approximately half their original height after EDTA treatment and remained stable following an additional cycle of locking and unlocking (Figure 3d). Comparisons of peak stress, elastic modulus, and energy dissipation across three different strains for cyclic loading in water revealed no significant differences between the first and second locked and unlocked states (Figure 3e–g). These data show that CoDE hydrogels retain their robust mechanical properties after two cycles of uncrosslinking and re‐crosslinking in a fully hydrated state.

**FIGURE 3:**
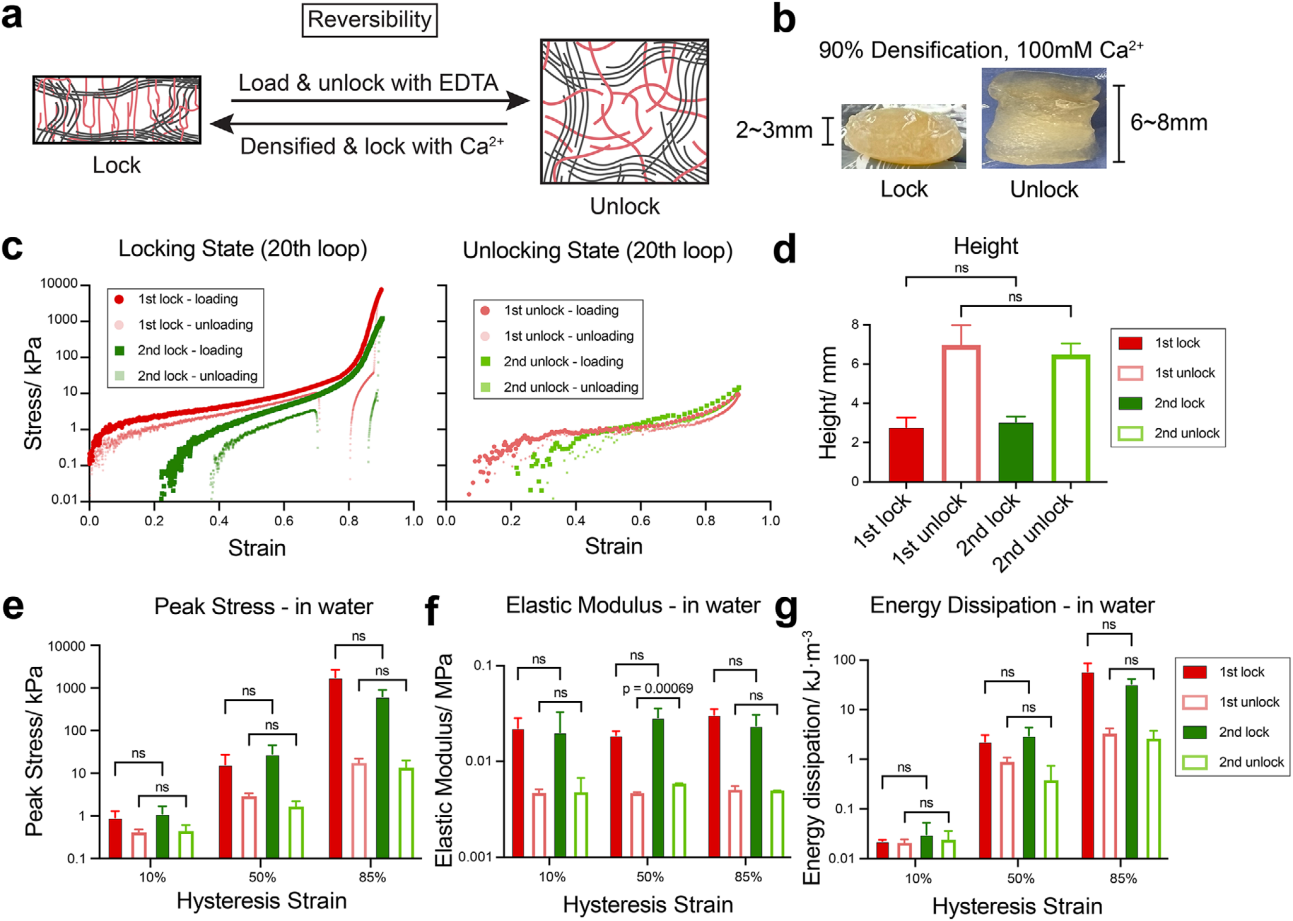
Reversible mechanical properties are demonstrated in repetitive locking and unlocking cycles of CoDE hydrogels. (a) Reversible locking–unlocking mechanism mediated by Ca^2^^+^ and EDTA. (b) Macroscopic demonstration of reversible height change. (c) 20th loop's stress–strain curves of CoDE hydrogels in aqueous environment under 85% hysteresis strain during the first and second locking and unlocking cycles. (d) Height of gels in mm after 20 loops of hysteresis tests under 85% strain (multiple unpaired t‐test, sample size = 3). Peak stress (e), elastic modulus (f), and energy dissipation (g) of hydrogels in water after 20 hysteresis loops of 10%, 50%, or 85% strain between first and second lock‐unlock cycles (multiple unpaired t test, sample size = 3, each sample value is the mean value of the last 10 loops). ns: no significant difference of two‐way ANOVA with post‐hoc test, *p* > 0.05. Error bars show the standard deviation of the mean.

The densified collagen structure of the CoDE hydrogel can be repaired after severing by re‐introducing alginate and Ca^2+^ crosslinking. Cylindrical CoDE samples were cut in half and sequentially soaked in 5 wt% alginate and 10 mm Ca^2+^ solutions to repair the severed samples. The samples were reformed into cohesive structures with no significant change in dimensions (Figure S3a,b). Tensile testing of the original and healed CoDE hydrogel tensile bars (Figure S3c,d) confirmed a partial recovery of mechanical properties under uniaxial tensile loading. The recovered strength and stretchability were lower than those of the original CoDE hydrogel (Figure S3e). These data suggest that the CoDE hydrogel may support reusability in certain low‐load applications and potentially enable real‐time repair of microdamage in calcium‐rich aqueous environments, such as marine settings.

The results suggest that the densified collagen network is the key load‐bearing structure of the CoDE hydrogel, while the alginate provides a dissipative network to prevent fracture and facilitates locking/unlocking and repair after severing. We hypothesized that the hierarchical structure of the densified collagen scaffold can evolve under repetitive loading to “train” the hydrogel and improve its mechanical properties. To quantify the intrinsic force generated by the densified collagen upon chemical unlocking and release of the alginate crosslinks, we used an 8 mm parallel‐plate rheometer geometry to measure the normal force (Figure 4a; Figure S4). This setup was designed for material‐level force characterization and does not represent an assembled robotic actuator or device. CoDE hydrogels were subjected to repeated compressive loading cycles on the rheometer. The z of the rheometer stage was held at the residual strain while the samples were treated with EDTA through the device to reverse the ionic crosslinking of the alginate, triggering the release of stored elastic energy and generating a measurable actuation force (Figure 4). Actuation force increased progressively with cycle number, reaching a maximum of 0.45 N for an 8 mm diameter specimen under 30 cyclic loadings (Figure 4b,c). This corresponds to ≈9 kPa axial stress, which is considered a large magnitude of force for an 8 mm hydrogel. In contrast, the residual strain also increased with increasing cycles but approached a plateau after repeated compression (Figure 4d), suggesting that the macroscopic densification of the collagen scaffold progressively reached a limit after 20 cycles. We interpret this difference as reflecting two distinct levels of structural evolution. The residual strain primarily reflects the phase transition from the rarefied, open‐pore state to the densified, pore‐collapsed state [14]. Force generation is also sensitive to the internal load‐bearing architecture of the densified collagen network. Second‐harmonic generation (SHG) imaging was performed to measure structural changes in the collagen fiber networks. Quantitative skeletonization analysis of maximum‐intensity projections from 3D SHG image stacks revealed a significant increase in junction density, defined as the number of fiber intersection nodes per image area, along with an increase in aligned fiber area fraction (Figure 4e). These findings indicate that, although further changes in the macroscopic residual strain become small after the scaffold approaches its densification limit, cyclic compression continues to promote fiber alignment [16] and increases inter‐fiber connectivity within the collagen scaffold. Such microstructural reorganization is consistent with more effective load transfer and greater elastic‐energy storage within the collagen scaffold, providing a mechanistic explanation for the continued increase in force upon chemical unlocking. These observations suggest that chemo‐actuation force is generated by elastic energy stored in the reorganized collagen scaffold, which is released by chelation of ions from alginate crosslinks.

**FIGURE 4:**
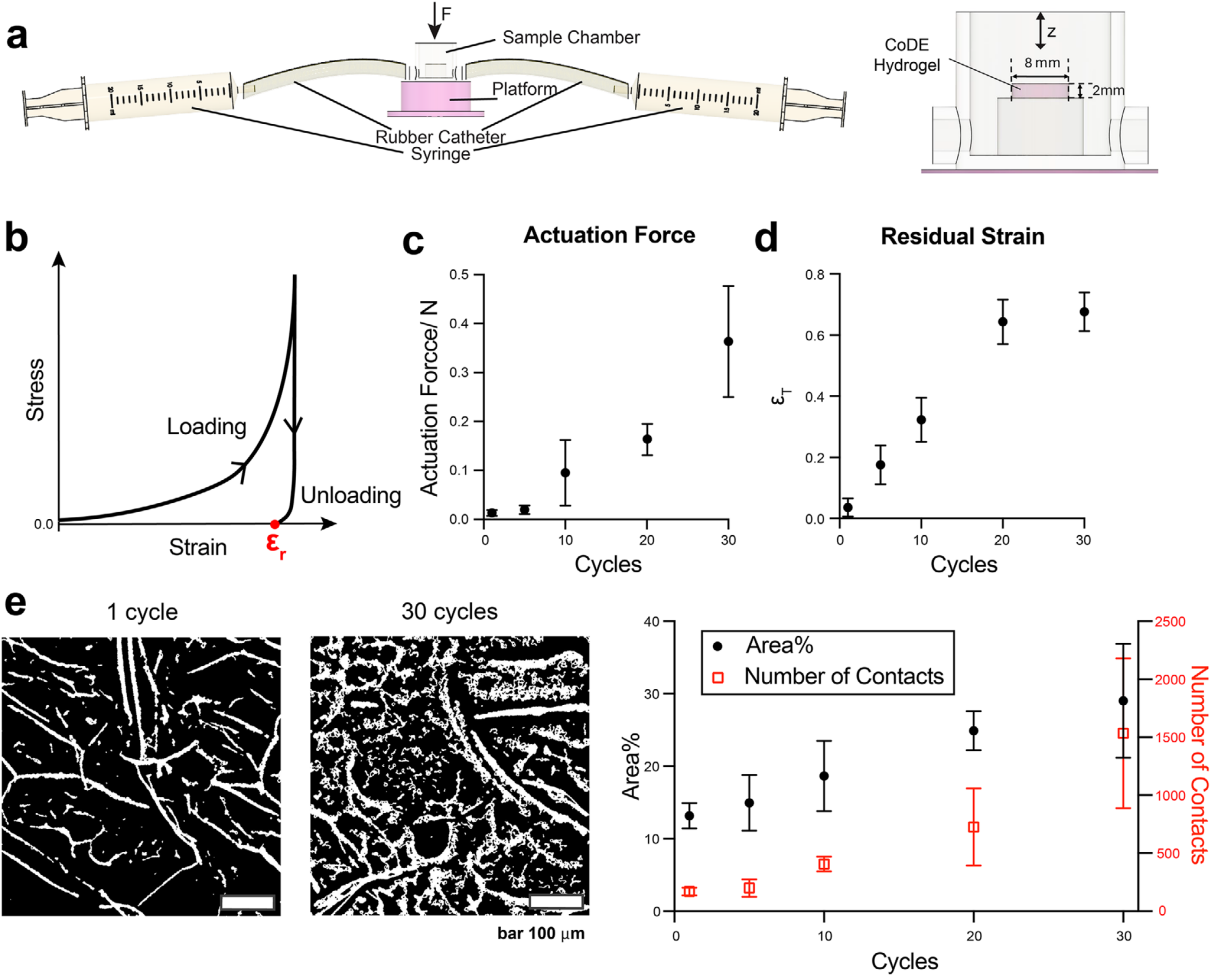
Cyclic compression reorganizes the CoDE structure, leading to increased fiber alignment and inter‐fiber connectivity, enhancing the actuation force, and shifting the residual strain. (a) Experimental setup for ionically actuated CoDE hydrogel with hydrogel geometry shown on the right. (b) Representative stress–strain curve showing nonlinear stiffening and residual strain (*ε*_r_). (c) Actuation force as a function of compression cycles, showing training‐dependent enhancement. (d) Residual strain (*ε*_r_) increases with cyclic compression and reaches a plateau at higher cycle numbers. (e) Microstructural evolution with training cycles: increased fiber alignment area and contact number (scale bar: 100 µm). Number of compression cycles = 1, 5, 10, 20 or 30 cycles, sample size = 3 gels per condition. Error bars show the standard deviation of the mean.

In contrast to the chemically triggered material‐level forces in Figure 4, the swimmer experiment in Figure 5 was designed to evaluate the mechanical durability and predictable deformation of CoDE under externally imposed cyclic loading. The CoDE hydrogel was integrated into a motor‐driven swimmer to evaluate its ability to sustain repetitive mechanical deformation after densification and ionic locking. The swimmer motion was externally driven in an aqueous environment, and this demonstration did not involve chemical triggering during operation. The swimmer was driven by a rotating‐disk mechanism that converted rotary motion into lateral oscillations, cyclically bending the hydrogel body (Figure 5a). The CoDE swimmer was not subjected to any cyclic mechanical loading before testing. The actuation system was mounted onto a rigid support frame and submerged in water during operation to mimic aquatic conditions (Figure 5b). All mechanical parts were designed via Fusion 360 and fabricated using 3D printing, with detailed dimensional specifications provided in Figure S5. The mounting plate (Figure S5a) provided the structural base for the assembly, ensuring stability during cyclic motion. The vertical support (Figure S5b) anchored the shaft and maintained the system's upright position during operation. The rotating disk (Figure S5d), whose diameter controlled the amplitude of the swimmer's movement, was centrally fixed to the shaft. A gripper (Figure S5e) connected the swimmer's head to the actuation system, providing secure attachment while enabling free‐end deformation. The system was electrically powered by a 9 V battery and controlled by a switch (Figure S5f).

**FIGURE 5:**
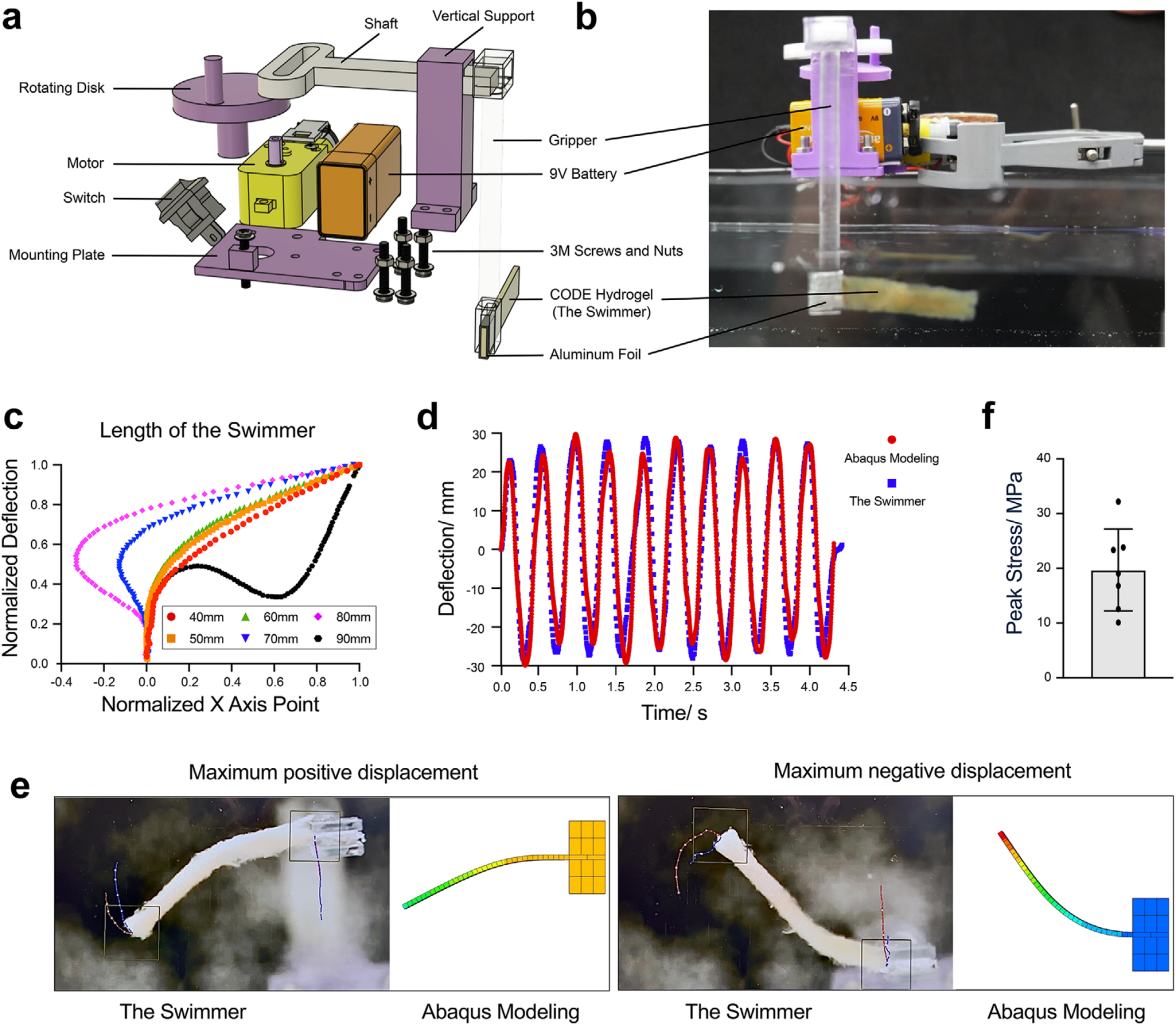
Experimental and computational analysis of the mechanical response of CoDE in a motor‐driven swimmer under cyclic loading. (a) Schematic Fusion 360 CAD diagram of the motor‐driven system used to impose cyclic deformation on the CoDE hydrogel swimmer. (b) Photograph of the experimental setup submerged in water, with the mounted swimmer visible. (c) Abaqus results: Normalized deflection profiles along the swimmer body for different swimmer lengths ranging from 40 mm to 90 mm. (d) Comparison of simulated swimmer deflection (red, Abaqus modeling) and experimental deflection (blue, tracked from high‐speed video) over ten cycles. The time‐deflection data show strong agreement in both amplitude and frequency. (e) Representative snapshots of swimmer configuration at maximum positive and negative displacements during cyclic motion. Left: experimental frames with motion‐tracked points; right: corresponding frames from Abaqus simulation. (f) Peak stress of 7 random samples punched from the swimmer. Each peak stress results from 90% compression, and each sample is a cylinder with a diameter of 8 mm and a height equal to the swimmer's thickness. Error bars show the standard deviation of the mean.

Parametric studies using numerical simulations in Abaqus/Explicit were performed to optimize the swimmer's geometry and actuation frequency to ensure stable, approximately linear variation of transverse deflection along the swimmer's length (Figure 5c; Figure S5g–k). In the simulation, the swimmer was represented by an effective homogeneous, isotropic material model to describe its mechanical response in the prepared state. As a result, shorter swimmers (40–60 mm) exhibited largely linear deflection along their length, while longer swimmers (≥70 mm) showed nonlinearity (Figure 5c and Figure S5g). Rotating disks with diameters ≤30 mm produced stable, nearly linear deformation profiles (Figure S5h,j). Motion periods of 0.4–0.7 s showed predominantly linear deformation, while shorter durations (0.2–0.3 s) resulted in under‐actuation and increased nonlinear deformation (Figure S5i,k). Based on the simulations, a configuration was selected for fabrication and testing: a swimmer length of 58 mm, an actuation amplitude of 30 mm (corresponding to the disk diameter), and a motion period of 0.45s.

Next, we compared the fabricated CoDE swimmer's experimental deflection during 10 swimming cycles, captured via high‐speed video, with finite‐element modeling results (Video S1). The simulation results aligned closely with the experimental data in both amplitude and frequency (Figure 5d). This agreement indicates that the measured deflection under the tested loading conditions can be reproduced without explicitly incorporating chemo‐mechanical coupling. This comparison evaluates the mechanical response in the prepared material state and does not test chemically triggered network reconfiguration. The CoDE swimmer withstood hundreds of swimming cycles, maintaining structural integrity and uniform mechanical strength (peak stress of 10–35 MPa) throughout the sample body (Figure 5f). These data suggest suitability for dynamic soft‐robotic applications in aquatic environments.

## 3 Discussion

The CoDE hydrogel exhibits programmable loading behavior spanning multiple orders of magnitude with stimulus‐dependent mechanical properties (Table S1). Although the range of the elastic modulus is similar to previous reports [17, 18], the biopolymer‐based CoDE system achieves a 160‐fold range in peak compressive stress (from kPa to MPa). The densified collagen scaffold and ionically crosslinked alginate synergistically reinforce the structural network and enhance peak stress, which aligns with the microstructural observation that compression induces a phase transition in the collagen scaffold [14], increasing fiber entanglement and inter‐fiber contact points before being locked by the alginate network. The densified collagen structure of the CoDE hydrogel, which sustains MPa loads, provides mechanical resilience beyond that of previous collagen‐alginate hydrogel systems [8]. One possible limitation of this hydrogel is that mechanical densification may expel some alginate from the porous collagen scaffold, thereby reducing Ca^2^^+^‐mediated shape fixation. The CoDE system is inherently biocompatible and biodegradable because it relies solely on collagen and alginate without reinforcement by synthetic polymers. We previously showed that the collagen‐densified structure can protect and maintain viable cells under large‐scale repetitive loading [14]. The CoDE system is highly versatile for soft applications and can be used to provide dynamic mechanical loading and matrix remodeling to guide cell fate [19, 20].

CoDE hydrogels exhibit reversibility and fatigue resistance, maintaining peak stress, elastic modulus, and energy dissipation across multiple lock‐unlock cycles under compressive strain up to 85%. Reversible chelation using EDTA and subsequent re‐crosslinking with Ca^2^^+^ mimics mutable collagenous tissues in sea cucumbers [12, 13]. The alginate network can be locked, unlocked, and re‐locked. The collagen scaffold provides shape‐memory properties [14] that enable recovery of mechanical properties. Chemical cycling over tens to hundreds of Ca^2^^+^/EDTA treatments will be required in future work to fully quantify the long‐term sustainability of repeated ionic reconfiguration. Severed gel samples regained cohesive structure and partially recovered their original tensile strength upon re‐exposure to alginate and calcium solutions. However, CoDE is not as tough or strong under tensile loading as synthetic systems, likely due to weak physical crosslinking. Free‐radical‐polymerized double‐network hydrogels can sustain large‐scale tensile deformation, but they also accumulate permanent damage under cyclic compression [9, 21, 22, 23].

Cyclic compressive loading mechanically “trains” the CoDE hydrogel, progressively increasing its cycle‐dependent actuation force to a maximum of 0.45 N for an 8 mm specimen. The calculated stress is on the same order of magnitude as the stress exerted by the longitudinal body wall muscle of the sea cucumber (≈20 kPa) [11, 12]. The actuation force can be attributed to the microstructural reorganization and increased junction density observed during cyclic compression. These findings align with recent full‐field molecular‐to‐macroscale biophysical imaging, which demonstrates that nonlinear stress actively induces transformations in collagen fibrillar organization and network disorder [24]. The alignment of biopolymer fibers is a broadly applicable mechanism for developing hydrogels capable of bearing high peak stresses [25]. This continued microstructural evolution of fibers within an approximately stabilized macroscopic geometry is consistent with enhanced load transfer and elastic energy storage, thereby allowing the chemically released force to continue increasing even after residual strain changes become small. Irreversible damage to the collagen network may also play a minor role, as was similarly observed in compressed fibrin networks [16]. While previous studies have shown that synthetic hydrogels can actuate in response to thermal or chemical stimuli, the ability to encode the mechanical history into a biopolymer network mimics the training response of mutable biological tissues. This capability to train a hydrogel's internal architecture to improve structural force output opens novel avenues for developing adaptive soft materials that respond predictively to their mechanical history for robotic soft interfaces [26]. Potential applications of CoDE include small‐scale soft robotic actuators, chemically triggered microgrippers, or stiffness‐switching biomedical devices that require moderate contractile stresses without electrical input.

The motor‐driven CoDE swimmer sustained hundreds of deformation cycles with kinematics that closely matched finite‐element simulations. This demonstration complements the material characterization by showing that the densified, ionically locked CoDE network can function as a compliant body under repetitive mechanical loading. The active material function of CoDE is demonstrated separately by the chemo‐mechanically triggered force generation in Figure 4. Previous soft robotic systems have successfully demonstrated wireless actuation for aquatic locomotion and grasping using light or magnetic stimuli [27]. However, soft robotic actuators often exhibit nonlinear, unpredictable mechanical behavior that is difficult to simulate. Experimental high‐speed visual tracking and Abaqus modeling data showed that the macroscopic bending and deflection of the CoDE hydrogel can be reliably modeled and controlled. This stable macroscopic performance is highly consistent with the bulk mechanical testing, which demonstrated the hydrogel's ability to maintain uniform high peak stresses (10–35 MPa) and resist fatigue‐induced fracture during repetitive loading. The bio‐friendly components of CoDE can be compatible with marine life [28]. The “digital twin” modeling approach presented here enabled in silico optimization to improve the design of hydrogel‐based machines for complex aquatic or biomedical environments.

## 4 Conclusions

A collagen‐alginate double‐network elastomeric (CoDE) hydrogel was developed with programmable loading behavior via collagen scaffold densification and ionic crosslinking of alginate. CoDE demonstrated fatigue resistance and reversibility, maintaining bulk mechanical performance across multiple chemically triggered lock‐unlock cycles without irreversible damage. Cyclic compression “trained” the internal collagen architecture by increasing fiber alignment and junction density, thereby increasing ionic actuation force. A bio‐inspired CoDE swimmer was driven to sustain repetitive, large‐scale deformation with dynamic kinematics accurately predicted by finite‐element computational modeling. The CoDE system is a versatile, mechanically trainable platform for hydrogel‐based soft‐material machines.

## 5 Materials and Methods

### 5.1 Materials

2‐[4‐(2‐Hydroxyethyl)piperazin‐1‐yl]ethanesulfonic acid (HEPES, 1 m) and Hanks’ Balanced Salt Solution (HBSS, 1x) were purchased from Gibco, FibriCol Type 1 Bovine Collagen Solution (10.2 mg mL^−1^) was purchased from Advanced BioMatrix, glutaraldehyde (GA) was purchased from TCI America, sodium hydroxide solution (NaOH, 10 m) and Ethylenediaminetetraacetic acid (EDTA) were purchased from Sigma‐Aldrich, calcium chloride (CaCl_2_) was purchased from Thermo Scientific, and PRONOVA UP VLVG sodium alginate was purchased from NovaMatrix. Deionized (DI) water was used for collagen cryogel synthesis. All chemicals used in the experiments were used as received, without further purification.

### 5.2 CoDE Hydrogel Fabrication

The fabrication of this collagen‐alginate double‐network elastomeric (CoDE) hydrogel involves two steps. First, the collagen‐based scaffold was prepared using the previously described method [14]. Briefly, the scaffold is formed by rapid vortex mixing of type‐I bovine collagen (5 mg mL^−1^) and GA crosslinker (0.15% w/v) in 20 mm HEPES buffer solution at pH 7.4. After casting in polypropylene molds, the samples are immediately transferred to ‐20°C for 24 h cryogelation and then 24 h lyophilization (‐80°C) to create a highly porous structure (porosity > 99.6%). The second network was incorporated into the collagen scaffold by soaking it in a 5 wt% alginate solution at 4°C for 24 h, allowing alginate to diffuse throughout the porous collagen network. The alginate‐infiltrated scaffold was then mechanically densified in a syringe using a displacement‐controlled procedure (Figure S1b). For each sample, the initial height (*h*_0_) was measured, and the sample was compressed to a prescribed final height (*h*_f_), corresponding to a target densification strain, *ε*_d_ = (*h*_0_−*h*_f_)/*h*_0_, of 0%, 50%, or 90%. The prescribed final height was used to control the densification state, rather than a prescribed compressive force. While the specimen was maintained at the final height, 10 mm or 100 mm CaCl_2_ solution was added into the syringe to crosslink the alginate network and “lock” the status. The CoDE was then soaked in the same concentration of CaCl_2_ solution for 24 h to ensure complete crosslinking and locking (Figure S1a).

### 5.3 Axial Compression and Hysteresis Experiments

All axial‐compression and hysteresis data from programmability tests were obtained using an Instron 5564 with a 2 kN load cell. The CoDE hydrogel samples were cylindrical, with a diameter of 8–10 mm and a height of 14–16 mm and were crosslinked in 10 mm or 100 mm CaCl2 solution with 0%, 50%, or 90% applied densification. Before testing, samples were pre‐conditioned with a 0.01 N preload to ensure the plate fully contacted the sample. Loading and unloading rates were set to 100 µm s^−1^, with a sampling speed of 10 ms s^−1^. All experiments began with the axial force readout reaching 0.01 N. All experimental data were obtained under compressive strain from 0% to 90% over 20 loops.

### 5.4 Reversibility Experiments

All CoDE hydrogel samples tested were cylindrical, with 90% densification, and for each locking step, samples were immersed in 100 mm CaCl_2_ solution for 24 h to allow Ca^2^^+^ diffusion and alginate ionic crosslinking. All hysteresis data on locking status (densified and crosslinked) for programmability tests were obtained in a 10 mm CaCl_2_ solution using an Instron 5564 with a 2 kN load cell. For the unlocking step, samples were immersed in EDTA solution for approximately 24 h to chelate Ca^2^^+^ and dissociate the alginate ionic crosslinks before subsequent testing or re‐locking. All “unlocked” (uncrosslinked) hysteresis data were obtained in DI water using a TA HR30 Discovery Hybrid Rheometer with a 20 mm parallel‐plate geometry at 25°C. Before testing, samples were pre‐conditioned with a 0.01 N preload to ensure the plate fully contacted the sample. Loading and unloading rates were set to 100 µm s^−1^ for the Instron and to the fast mode for the rheometer, with a sampling speed of 10 ms s^−1^. All experiments began with the axial force readout reaching 0.01 N. Experimental data were obtained from each CoDE hydrogel undergoing 2 locking‐unlocking cycles and tested under compressive strains of 0%, 50%, or 85% for 20 loops. All tests were first performed on “locked” CoDE hydrogels and then uncrosslinked with EDTA solution overnight before obtaining data on “unlocked” hydrogel.

### 5.5 Actuation Experiments

All CoDE hydrogel samples tested were at less than 90% densification and crosslinked in a 100 mm CaCl_2_ solution. To ensure the uniformity of the samples’ shape, a Miltex Biopsy Punch 8 mm was used to cut CoDE hydrogels into cylinders, with the height equal to the hydrogel thickness. All actuation data were obtained using a TA HR30 Discovery Hybrid Rheometer with an 8 mm parallel plate at 25°C. Before testing, samples were pre‐conditioned with a 0.05 N preload to ensure the plate fully contacted the sample. Loading and unloading rates were set to 10 µm s^−1^ at a linear rate, with sampling speed set to fast mode. All experiments began with the axial force readout reaching 0.05 N. Experimental data were obtained from each CoDE hydrogel tested under compressive strain from 0% to 85% with 1, 5, 10, 20, and 30 cycles. After the repetitive loadings were completed, the samples were held at the phase‐transition strain under stress‐relaxation conditions until the axial force stabilized. EDTA was added to the testing platform, which then soaked the CoDE hydrogel sample, and the actuation force was immediately measured from the change in axial force.

### 5.6 Abaqus Modeling

Numerical simulations of the swimmer's motion in a fluid environment were conducted using Abaqus/Explicit 2022. The model consists of three primary parts: a cubic Eulerian body representing the water environment, a rigid gripper modeled as a solid part, and a deformable swimmer modeled as a shell structure. The Eulerian domain is a cube measuring 200 mm × 200 mm × 200 mm that encloses both the gripper and swimmer to simulate fluid confinement. This part was defined with properties matching water: density of 1000 kg m^−^^3^, bulk speed of sound *C*_0_ = 1480 m s^−1^, and Equation of State (EOS) parameters configured as US‐UP form with a uniform distribution. EOS constants were set to *S*_0_ = 1 × 10^−^^8^ and *γ*_0_ = 0, assuming ideal compressibility.

The gripper was modeled as a rigid body with dimensions matching those of the physical 3D‐printed device. Material properties were based on polylactic acid (PLA), with a density of 1250 kg m^−^^3^, Young's modulus of 3.5 GPa, and Poisson's ratio of 0.36. The swimmer's body, made of a highly porous scaffold with over 99% water content, was assumed to have the same density as water (1000 kg m^−^^3^). The swimmer was modeled as a homogeneous isotropic shell with a Young's modulus of 14.5 kPa, a Poisson's ratio of 0.1, and a shell thickness of 2 mm. The swimmer was modeled as a homogeneous, isotropic material to describe its response to externally imposed cyclic mechanical loading. Chemical triggering and changes in network connectivity were not included in the model. Its geometric dimensions were set to 58.44 mm in length and 11 mm in width, based on physical measurements.

Following a mesh convergence analysis, a 1 mm mesh size was used for both the Eulerian cube and the gripper. For the swimmer, structured rectangular shell elements with in‐plane dimensions of approximately 2 mm × 0.5 mm were used to accurately capture its flexible deformation, and the selected seed size met the mesh convergence threshold. The elongated 2 mm × 0.5 mm element shape was chosen to align with the swimmer's high aspect‐ratio geometry. Therefore, the selected mesh achieved a balance between numerical accuracy and computational cost. It should be noted that the shell thickness (2 mm) represents a section property corresponding to the physical specimen thickness, whereas the mesh dimensions (2 mm × 0.5 mm) describe the in‐plane discretization of the shell surface. A reference point (RP‐1) was created using the Datum feature to control the gripper's displacement input. Each component was organized into corresponding sets for boundary conditions and interaction definitions. The swimmer surface was partitioned into two segments: tm, representing the portion connected to the gripper, and ts1, representing the freely deforming region, thereby enabling accurate assignment of constraints and interactions.

Boundary conditions were applied using step‐based time controls. BC‐1 defined the Eulerian inflow conditions, while BC‐2 controlled the motion of the gripper. A periodic amplitude was defined with a circular frequency of 13.9626 rad s^−1^, consistent with the physical rotation of the motor's cam‐based actuation system. Another periodic amplitude input was used to simulate gripper movement along the U1 (X) direction with a peak displacement of 15 mm, corresponding to the radius of the rotating disk in the physical setup. Using circular frequency ensures that the sinusoidal input profile reflects continuous harmonic motion, and 10 complete cycles were defined in Abaqus to replicate the experimental video duration.

The simulation job was submitted and solved using explicit dynamic analysis. The resulting outputs were focused on nodal displacement fields, specifically U (total displacement magnitude) and U1 (X‐directional displacement). Two nodes on the swimmer were selected for post‐processing: one at the free tail end and the other at the interface with the gripper. The difference in their U1 displacements was computed to quantify the swimmer's bending deflection throughout the actuation cycles. The displacement‐time data were exported for further analysis and comparison with experimental motion tracking data.

### 5.7 Particle Tracking

Particle motion tracking was performed in Blender (version 4.4), an open‐source 3D creation and compositing suite with a built‐in motion‐tracking tool. Videos of the motor‐driven CoDE swimmer were recorded at 120 frames per second using a FUJIFILM X‐T3 camera equipped with a FUJINON XF80mmF2.8 R LM OIS WR Macro lens. The videos were then imported into Blender for analysis. Two key features were manually selected for tracking in the “Marker” option: the swimmer's tail tip and the head connection point, defined as the midpoint at the interface between the swimmer and the gripper. Frame‐by‐frame motion tracking was conducted for both markers using the “Location” motion model under the “Keyframe” match setting, with a pattern size of 21 pixels and a search size of 71 pixels. “Prepass” and “Normalize” options were enabled to improve track stability and compensate for brightness variation. Upon completion of the tracking process, the Y‐coordinates of both feature points were exported as time‐series data. As the swimmer initially aligns horizontally, the Y‐values at both points are identical. Therefore, the difference in Y‐coordinates over time corresponds to the relative deflection of the swimmer. The Blender video‐plane Y direction corresponded to the transverse X direction in the Abaqus simulation. The relative deflection was converted from image coordinates to millimeters using a spatial calibration factor.

### 5.8 Data Analysis and Statistics

All mechanical, hysteresis, reversibility, and actuation experiments were performed with a sample size of *n* = 3 independent hydrogel specimens. Experimental data are presented as the mean values, with error bars representing the standard deviation of the mean. For long‐term continuous cyclic loading tests, individual sample values were reported as the mean of the final 10 cycles. Data graphing and statistical analyses were conducted using GraphPad Prism software. To determine statistical significance between experimental groups, multiple unpaired t‐tests and a two‐way ANOVA with post hoc tests were used. Statistical significance was defined as a *p*‐value < 0.05, with non‐significant differences denoted as “ns” (*p* > 0.05). Linear regression analysis was reported with *R* ^2^ values and *p*‐values. Frame‐by‐frame particle‐motion tracking of the bio‐inspired swimmer was performed in Blender to extract absolute deflection data over time. Microstructural junction density and aligned fiber fraction were evaluated via quantitative skeletonization analysis of maximum‐intensity projections from 3D second harmonic generation (SHG) image stacks. Additionally, the graphical abstract was edited utilizing NotebookLM and Gemini Pro, and Grammarly was utilized for text editing.

## Author Contributions

Y.L., X.H., and K.H.V. contributed to the conceptualization, data curation, writing, and review & editing of this manuscript. YL and XH contributed to the investigation, data analysis, and visualization. P.K.P. and K.H.V. contributed to supervision and mentoring. K.H.V. and P.K.P. provided funding support for the project.

## Conflicts of Interest

The authors declare no conflicts of interest.

## Supporting information




**Supporting File 1**: advs78047‐sup‐0001‐SuppMat.pdf.

**Supporting File 2**: advs78047‐sup‐0002‐VideoS1.mp4.

## Data Availability

All data used to generate figures for this manuscript will be made available prior to publication in a Dryad public repository at https://doi.org/10.5061/dryad.f1vhhmhb9.
