# Supplementary material for: Sea Cucumber‐Inspired Double‐Network Biopolymer Hydrogels for Programmable Soft Machines
Source: Adv Sci (Weinh). 2026 Oct 11:e78047. Online ahead of print. doi: 10.1002/advs.78047 (PMC13655269; doi:10.1002/advs.78047)
Supplement: Supplementary file 1 [file ADVS-9999-e78047-s002.pdf]

Supplementary Information

Sea Cucumber-Inspired Double-Network Biopolymer Hydrogels for Programmable Soft Machines

Luo, Yan[1], He, Xiao[1], Purohit, Prashant K.[1], Vining, Kyle H.[2, 3, 4*]

1 Department of Mechanical Engineering and Applied Mechanics, School of Engineering and Applied Science, University of Pennsylvania, Philadelphia, PA, United States

2 Preventive and Restorative Sciences, School of Dental Medicine, University of Pennsylvania, Philadelphia, PA, United States

3 Department of Materials Science and Engineering, School of Engineering and Applied Science, University of Pennsylvania, Philadelphia, PA, United States

4 Center for Innovation and Precision Dentistry, University of Pennsylvania, Philadelphia, PA, United States

*Corresponding Author, viningk@upenn.edu

Supplemental Methods

1. Compression Rate Comparison Test - All axial compression and hysteresis data from compression rate comparison tests were obtained using a TA HR30 Discovery Hybrid Rheometer with a 20mm parallel plate geometry at 25°C. Samples were cylindrical, with a diameter of 8-10mm and a height of 14-16mm. Before testing, samples were pre-conditioned with a 0.05N preload to ensure the plate fully contacted the sample. Loading and unloading rates were set to 100 μm/s or 200 μm/s linear rate, with the sampling speed set to fast mode. All experiments begin with the axial force readout reaching 0.05N.

2. Tensile Test - CoDE hydrogel samples were fabricated into tensile bars with 90% densification and crosslinked in a 100 mM Ca^{2+} solution. All tensile test data were obtained using an Instron 5564 with a 2kN load cell. The loading crosshead speed was set to 1 mm/min, with a sampling speed of 10 pts/s. All experiments end when the axial force readout reaches 0N.

Table S1. Summary of CoDE Hydrogel Properties.

Stimulus	Network Changes	Mechanical Properties
Compression	Collagen scaffold densification	Increased peak stress
Ca^{2+} treatment	Alginate ionic crosslink (locking)	Enhanced energy dissipation
EDTA treatment	Alginate uncrosslink (unlocking)	Reversible soft state
Cyclic loading	Collagen fiber alignment & contacts	Increased actuation force

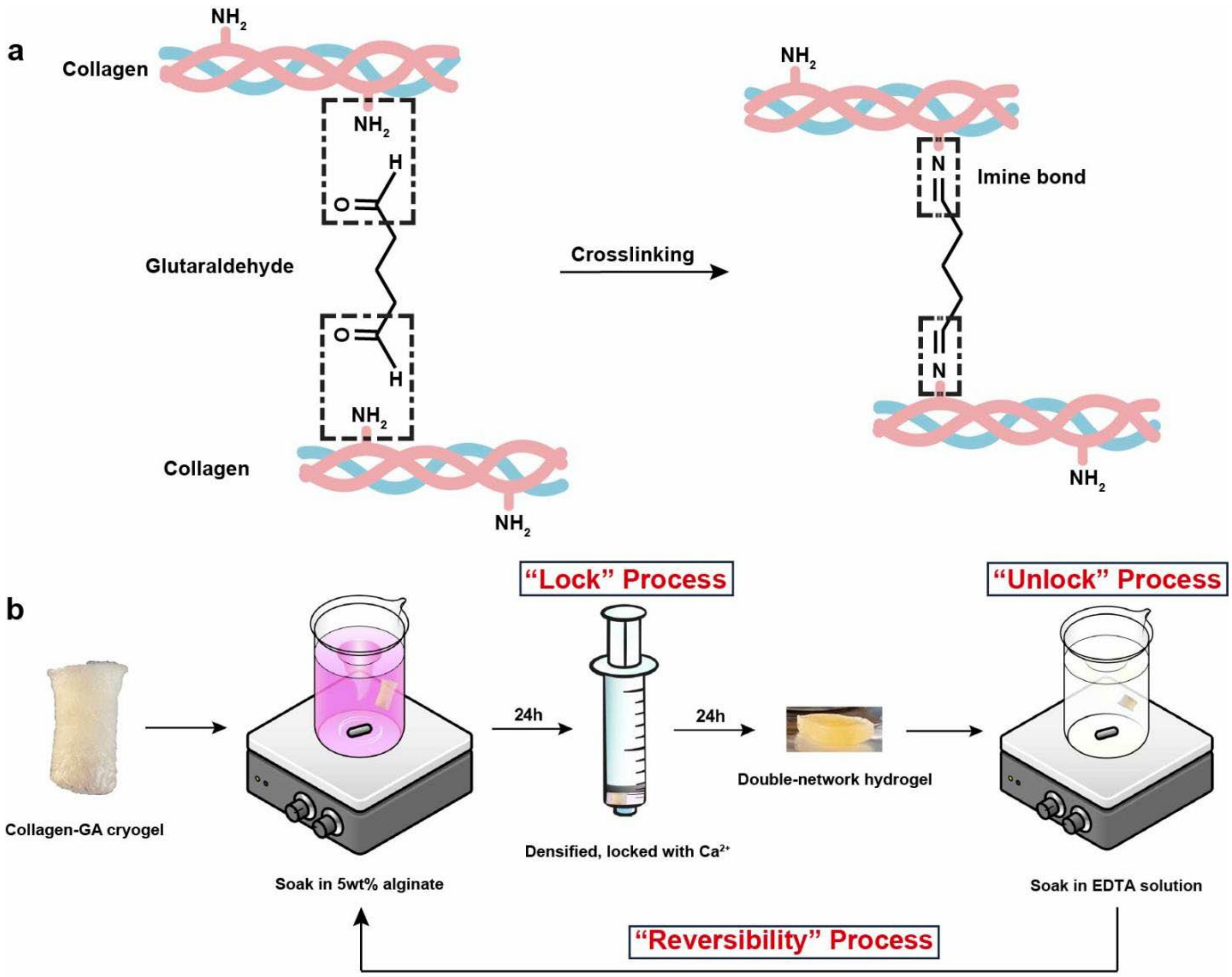

Figure S1. Chemical crosslinking mechanism of collagen-GA cryogel and reversible double-network hydrogel formation. (a) Chemical crosslinking mechanism of collagen-GA cryogel. **(b)** The reversible synthesis process of turning collagen-GA cryogel into a collagen-alginate double-network hydrogel.

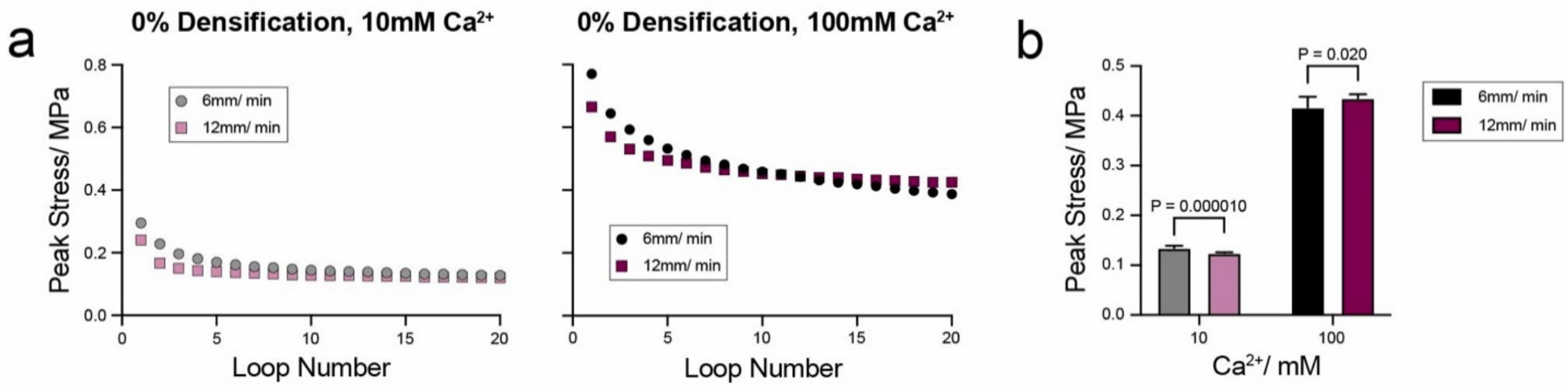

Figure S2. Compression rate's minimal influence on CAD hydrogels' peak stress results. **(a)** Cyclic compressions test 0% densified and 10 mM (left) and 100 mM (right) Ca^{2+} crosslinked collagen-alginate hydrogels under 20 hysteresis loops of strain from 0 to 0.9 and 2 different compression rates (6 mm/min and 12 mm/min). **(b)** Statistical analysis of peak stress for samples crosslinked with the same Ca2+ concentration but tested under different compression rates.

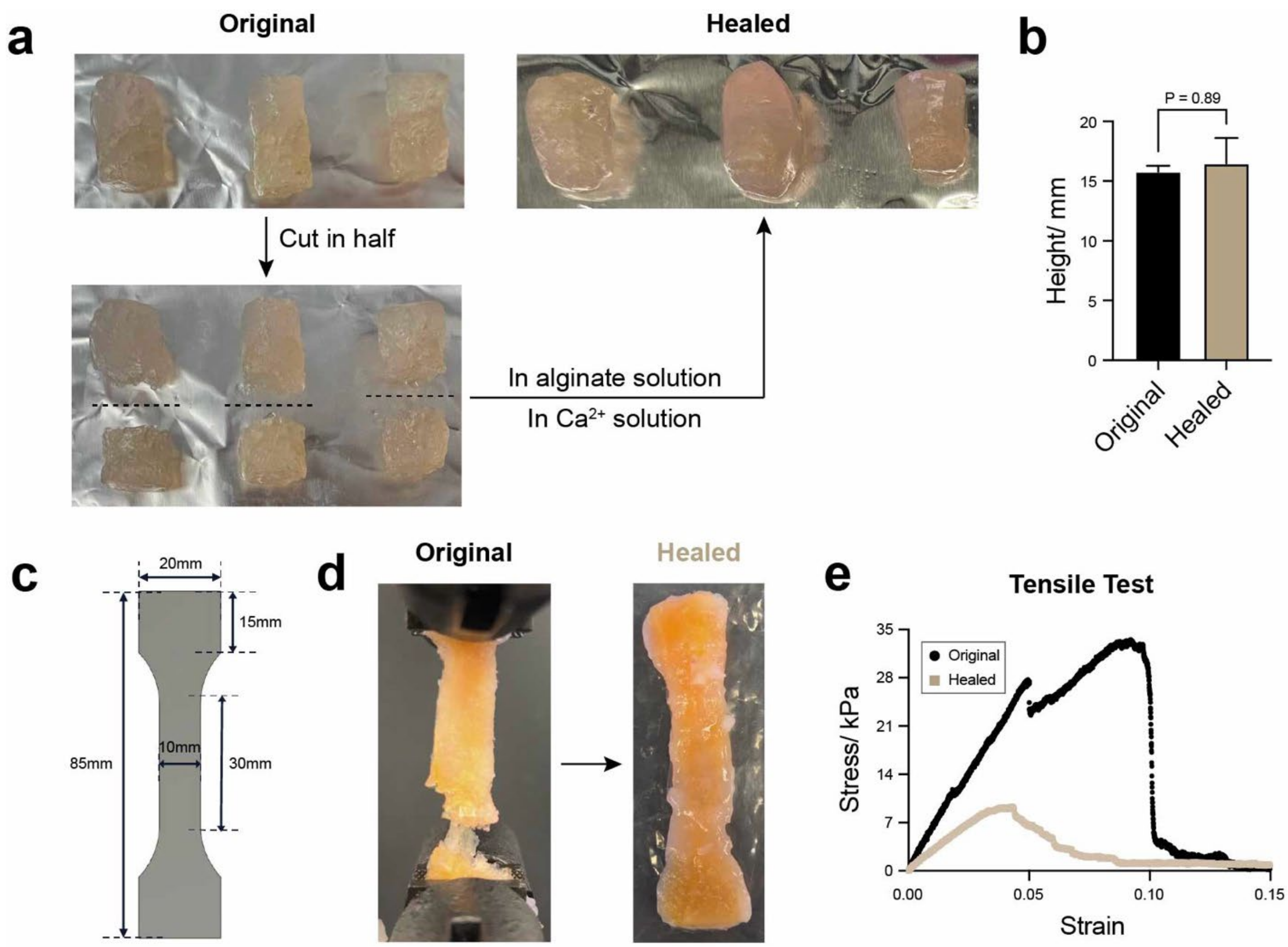

Figure S3. Self-healing property of the collagen–alginate double-network hydrogel. (a) Photos of the self-healing process for the three hydrogel samples. **(b)** Statistical results of sample height before and after self-healing. **(c)** AutoCAD design of the tensile bar. **(d)** Photos of the

tensile bar of hydrogel breaking during the tensile test (left) and after self-healing (right). **(e)** Tensile test data of hydrogel before (black) and after (brown) self-healing.

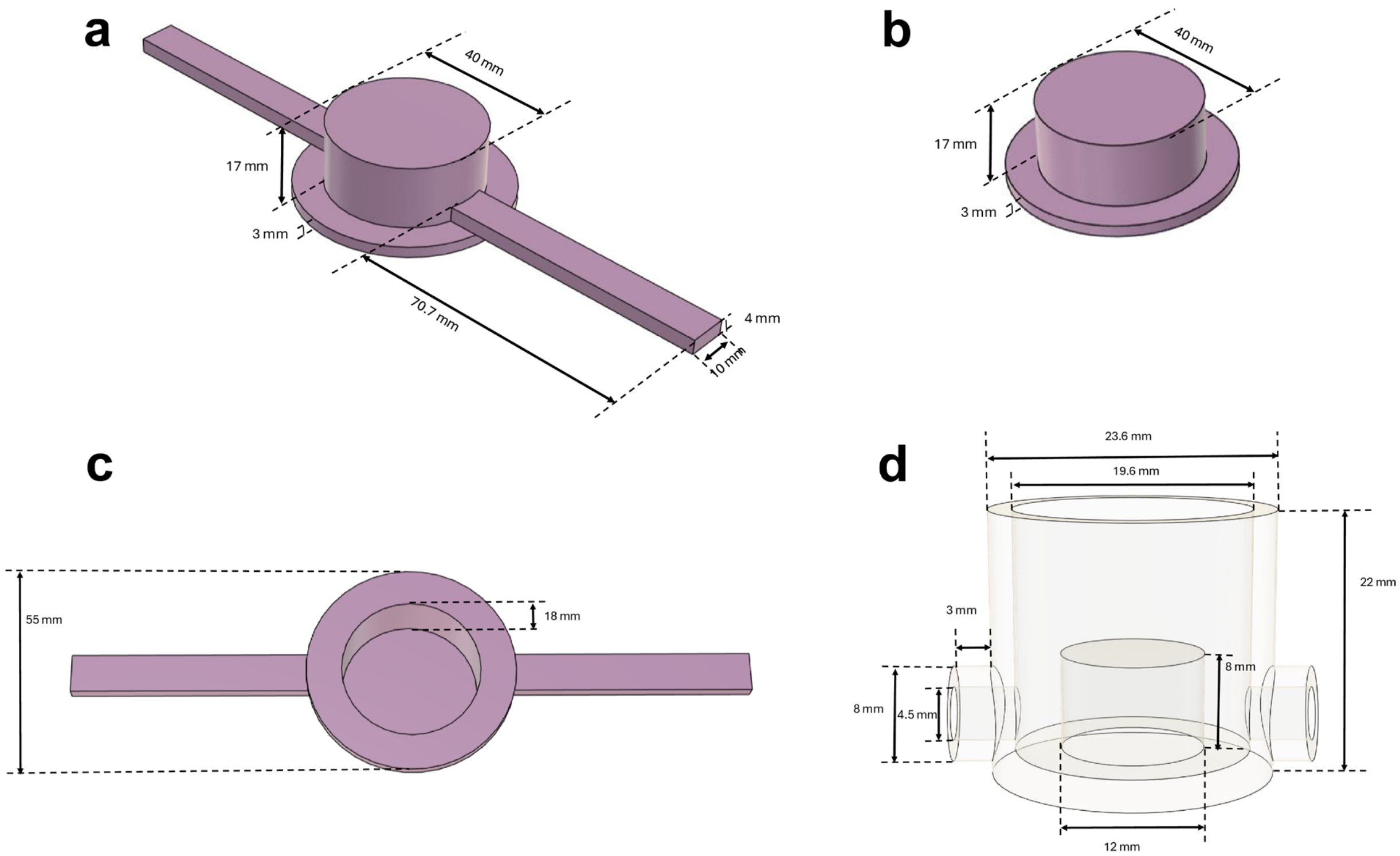

Figure S4. AutoCAD drawings for ionically actuated CoDE hydrogel geometry. (**a**) Assembly, (**b**) the central housing, (**c**) bottom view, and (**d**) gel chamber.

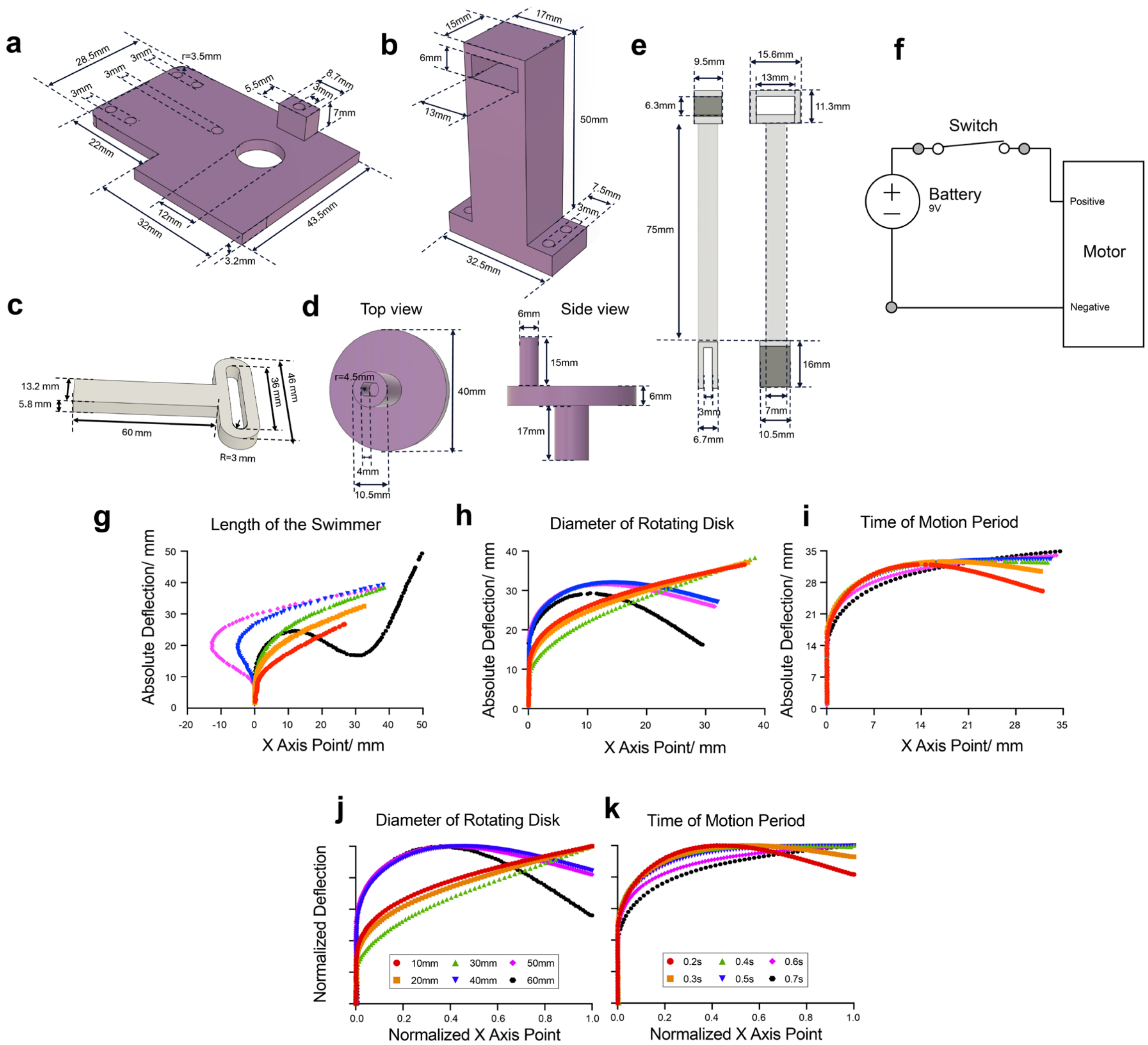

Figure S5. AutoCAD drawing, circuit design, mechanical performance, and prediction of the battery-actuated soft swimmer. AutoCAD drawing and dimensions for **(a)** the mounting plate, **(b)** the vertical support, **(c)** the shaft, **(d)** the rotating disk, and **(e)** the gripper. **(f)** Electrical circuit diagram showing the wiring configuration for the 9 V battery-powered motor and the actuation device switch. Abaqus modeling results: Absolute deflection profiles **(g)** along the swimmer body for different swimmer lengths (40 mm to 90 mm), **(h)** for different rotating disk diameters (10 mm to 60 mm), corresponding to varying actuation amplitudes, and **(i)** for different motion periods per cycle (0.2 s to 0.7 s). **(j)** Normalized deflection profiles of rotating disk diameters representing varying actuation distances, and (**k**) motion periods ranging from 0.2 s to 0.7 s per cycle.

Video S1. Video recording and particle tracking of CoDE hydrogel swimming strokes.
